# The Current Strategies in Controlling Oral Diseases by Herbal and Chemical Materials

**DOI:** 10.1155/2021/3423001

**Published:** 2021-08-21

**Authors:** Mohammad Nima Motallaei, Mohsen Yazdanian, Hamid Tebyanian, Elahe Tahmasebi, Mostafa Alam, Kamyar Abbasi, Alexander Seifalian, Reza Ranjbar, Alireza Yazdanian

**Affiliations:** ^1^Research Center for Prevention of Oral and Dental Diseases, Baqiyatallah University of Medical Sciences, Tehran, Iran; ^2^Science and Research Branch, Islamic Azad University, Tehran, Iran; ^3^Department of Oral and Maxillofacial Surgery, School of Dentistry, Shahid Beheshti University of Medical Sciences, Tehran, Iran; ^4^Department of Prosthodontics, School of Dentistry, Shahid Beheshti University of Medical Sciences, Tehran, Iran; ^5^Nanotechnology and Regenerative Medicine Commercialization Centre (Ltd), The London Bioscience Innovation Centre, London, UK; ^6^School of Dentistry, Baqiyatallah University of Medical Sciences, Tehran, Iran; ^7^Department of Veterinary, Science and Research Branch, Islamic Azad University, Tehran, Iran

## Abstract

Dental plaque is a biofilm composed of complex microbial communities. It is the main cause of major dental diseases such as caries and periodontal diseases. In a healthy state, there is a delicate balance between the dental biofilm and host tissues. Nevertheless, due to the oral cavity changes, this biofilm can become pathogenic. The pathogenic biofilm shifts the balance from demineralization-remineralization to demineralization and results in dental caries. Dentists should consider caries as a result of biological processes of dental plaque and seek treatments for the etiologic factors, not merely look for the treatment of the outcome caused by biofilm, i.e., dental caries. Caries prevention strategies can be classified into three groups based on the role and responsibility of the individuals doing them: (1) community-based strategy, (2) dental professionals-based strategy, and (3) individual-based strategy. The community-based methods include fluoridation of water, salt, and milk. The dental professionals-based methods include professional tooth cleaning and use of varnish, fluoride gel and foam, fissure sealant, and antimicrobial agents. The individual-based (self-care) methods include the use of fluoride toothpaste, fluoride supplements, fluoride mouthwashes, fluoride gels, chlorhexidine gels and mouthwashes, slow-release fluoride devices, oral hygiene, diet control, and noncariogenic sweeteners such as xylitol. This study aimed to study the research in the recent five years (2015–2020) to identify the characteristics of dental biofilm and its role in dental caries and explore the employed approaches to prevent the related infections.

## 1. Introduction

Oral cavity provides an environment that leads to the colonization and growth of an extensive range of microorganisms. Bacteria are the most prevalent of them. The highest bacterial accumulation is found as a biofilm on the tooth surface (dental plaque). The loss of mucosal surface reduces the microbial load on the mucosal surfaces [1, 2]. These microbes normally exist on all oral surfaces and are necessary for the normal physiological growth of the oral cavity [3]. Static microflora contributes to the host health by preventing the establishment and colonization of exogenous microorganisms and potential pathogens and regulating the inflammatory response of the host to oral commensal bacteria [4]. The oral bacterial population with almost 1000 species is very complicated [5]. The clinical treatment of caries usually deals with the restoration of hard tissues considering the functional and aesthetic needs. However, this does not treat the illness but treats its outcomes. In clinical settings, a common method for the management of dental caries is the elimination of contaminated tissues to prevent the further progress of the disease. The areas surrounding the restoration gradually undergo caries, so a small restoration must be substituted by a greater restoration and dental tissues are increasingly eliminated. Repetition of this cycle leads to extensive loss of dental tissue, pulpal involvement, and finally tooth loss [6]. Admittedly, an inadequate understanding of dental caries as a biological process leads dentist to such a problem. Considering the developments in genetics, particularly molecular biology, findings make it possible for us to replace the old paradigms with new ways of caries prevention and treatment. Dental decay is caused by an ecological imbalance in mouth microflora. These imbalances can be influenced by environmental and biological reasons. Dental caries can be controlled by managing the above mentioned changes [7, 8]. In this review, we aimed to study the recent research to identify the characteristics of dental biofilm and its role in dental caries and also explore the employed methods to prevent the related infections.

## 2. Caries and Its Stages

Dental caries is a chronic and preventable disease caused by dental biofilm activity. This disease is multifactorial and is initially caused by an imbalance of mouth microflora as a result of existence of carbohydrates on the tooth. Caries is characterized by local demineralization of the teeth and loss of dental structure. Some biofilm bacteria metabolize the existing carbohydrates and produce acids. These acids can reduce the pH and reach lower than the critical limit (5.5 for enamel and 6.2 for dentine) if they remain in the biofilm for a long time. This acidic environment affects the biofilm composition and tooth surface. Therefore, acidogenic and acidophilic bacteria increase, which results in a more acidic environment. Calcium and phosphate are removed from the tooth surface, as a result of which demineralization occurs due to the loss of minerals. If pH returns to its normal level, calcium and phosphate can return to the tooth structure and cause remineralization [9]. Dental caries is created on the surface and subsurface areas of the tooth following a dynamic trend of demineralization and remineralization. These events occur several times over time and are influenced by several factors like the type and number of biofilm microorganisms, diet, oral hygiene, genetics, tooth anatomy, fluoride consumption, and salivary content and composition. These factors are specific to each individual and vary from tooth to tooth and from place to place [9]. As demineralization continues, primary caries occurs, a lesion with no cavities called white spot lesion (WSL) on enamel. Most primary caries can be terminated or remineralized; i.e., they are reversible. Dental caries becomes cavitated as it progresses. Cavitated lesions often need restorative intervention and are irreversible. Moderate lesions are lesions that have not extended the internal one-third of the dentin, and advanced or deep lesions are those that have extended the internal one-third of the dentin [9].

## 3. Dental Plaque

Dental plaque is a soft and sticky layer accumulated on the tooth surface. Dental plaque, also called biofilm, mostly consists of bacteria and their products, extracellular matrix, and water. Biofilm is not sticky food debris or accidental accumulation of opportunistic microorganisms; it is the accumulation of a series of organized events. The formation of dental plaque involves the formation of the pellicle, initial attachment, and plaque maturity [10]. Plaque forms as follows: a clean tooth surface is immediately exposed to the salivary byproducts, gingival crevice fluid (GCF), and some compounds resulting from bacteria. These products are absorbed by the surface with a negatively charged hydroxyapatite, creating a layer called acquired pellicle. The dental pellicle is covered with positively charged molecules, which contain more than 180 proteins, peptides, and glycoproteins such as proline-rich proteins, histidine-rich proteins, phosphoproteins (e.g., statherin), creatine, mucin, and other molecules that act as the binding sites for bacteria [10]. Some bacterial products like glycosyltransferases and glucans are also found in the pellicles. Interestingly, the main compound of a pellicle is stable in different areas of the oral cavity and among individuals. The bacteria attached to the tooth surface are not directly connected to the enamel; they are bound to the enamel through pellicle [10]. Primary bonding is initiated a few minutes after brushing the colonization. A cell wall with negatively charged bacteria facilitates its connection to the positively charged receptors of the pellicle. The early stages of transfer and interference with the surface are similar and nonspecific for all bacteria. Specific involvement of the binding molecules of the bacterial cell wall and pellicle receptors determines whether the bacteria remain in contact with the surface or not. Only a small part of oral bacteria can bind to the pellicle receptors, which constitutes the most common biofilm bacteria over a short time after tooth cleaning. During the early hours, streptococci form over 60–80% of biofilm bacteria. These bacteria along with *Haemophilus*, *Neisseria*, *Actinomyces*, and *Veillonella* species are known as primary colonizers. They initially create a wide range of nonspecific and reversible Van der Waals connections with pellicle (more than 50 nm). Then, they make stronger and irreversible short-range connections (10–20 nm) with pellicle receptors by their specific surface molecules [10].

Streptococci create different adhesion mechanisms. They have different adhesion mechanisms such as products of glycosyltransferases, glucan-binding proteins, and pili, while other bacteria such as actinomycetes bind to the surface using their fimbriae [11]. These bacteria provide new adhesion sites for other bacteria and change the environment with their metabolic activity, thereby affecting the biofilm viability (e.g., reducing the ambient oxygen) [11]. Plaque matures as follows: the primary colonizing bacteria bound to the tooth surface provide new receptors for the bacteria to help induce coadhesion via binding. The bacteria of different species or even strains of a species can specifically bind to specific bacteria. *Fusobacterium* coaggregates with all oral bacteria, while *Veillonella*, *Capnocytophaga*, and *Prevotella* coaggregate with streptococci or actinomycetes. Most of this adhesion between various bacteria is performed through lectin-like receptors (proteins that identify the carbohydrates). Thus, they can be inhibited by lactose or other galactosidase or amino acids like L-arginine [10, 12]. Secondary colonizers like *P.Int*, *FN*, *Prevotella loescheii*, *Capnocytophaga*, and *PG* cannot bind to the clean tooth surface but can bind to bacteria in the dental plaque. Some specific structures in dental biofilm such as corn cob and test-tube brush are created due to the adhesion of cocci to filamentous bacteria. In this stage of plaque maturity, the bacteria secret extracellular polysaccharide, which constitutes the biofilm scaffold [10, 13]. If the dental plaque remains for about 7 days, it provides a favorable environment for the colonization of some aerobic Gram-negative bacteria, which are called secondary colonizers, including *PG*, *Aggregatibacter actinomycetemcomitans*, and *Treponema denticola* [10, 13].

## 4. Dental Biofilm

A healthy dental biofilm is mostly composed of commensal nonpathogenic microbes. These microbes are not completely independent and are regularly linked with each other and host tissues like gingiva even in a healthy state. The host provides the surfaces for colonization, and beneficial bacteria prevent the colonization of pathogenic bacteria [4, 14]. The advantages of this connection are manifested in conditions such as antibiotic sore mouth when inhibition of normal flora leads to the growth of opportunistic pathogens [4]. Studies have shown that commensal bacterial species such as *S. salivarius*, *S. sanguis*, and *Atopobium parvulum* can induce biofilm health-related conditions. Nevertheless, further research is needed to explore this issue. It has been shown that *S. salivarius* inhibits the quorum sensing process and mutans biofilm formation, which results in its anticaries properties [15–17]. The commensal bacteria in biofilm are involved in the immune system development. They do this by presenting diverse antigens to the host immune system. The commensal bacteria create a cascade of signals that induce the host resistance, while pathogenic bacteria induce severe inflammation in the host. Hence, proinflammatory cytokines are produced in a low number in oral epithelial cells, which induces the expression of E-selection in vascular endothelial tissues and produce IL8 [10]. The commensal bacteria induce the innate immune response of the host, which strategically juxtaposes the neutrophils with subgingival bacteria and junctional epithelium. The main bacteria with the highest variation in the oral cavity include *S*, *Staphylococcus*, *Peptostreptococcus*, *P*, *Haemophilus*, *Veillonella*, *Leptotrichia*, *Treponema*, *Propionibacterium*, *Actinomyces*, *Fusobacterium*, *Corynebacterium*, *Eikenella*, *Gemella*, *Granulicatella*, *Rothia*, *Porphyromonas*, *Capnocytophaga*, *L*, *Neisseria*, and *Eubacteria* [18–20]. Oral health depends on the conservation of its normal microflora. A disease occurs once the species are imbalanced and pathogens become dominant. Oral health and disease are dynamic processes in which the ecology of communities is a determinant factor, not an organism. Understanding the meaning of and identifying the molecular changes among the disease and health conditions provide the clinicians with the ability to diagnose and reverse the disease in early stages [21].

## 5. Bacterial Groups in the Caries Process

The newest molecular biology methods of microorganisms related to caries are *B. dentium*, *B. adolescentis*, *SM*, Scardovia wiggsiae, *B. longum*, *Selenomonas* spp., *P.* spp., and *L.* spp. [16]. Studies have indicated that early streptococci on the newly cleaned tooth surface mostly include *S. sanguinis*, *S. oralis*, and *S. mitis* strains, and the content of their mutans is only 2% or less. It has also been shown that most primary colonizers belong to the *S. mitis* group. The amount of microflora actinomycetes increases over time so that the smooth surfaces of most bacteria in the mature plaque include actinomycetes and nonmutans streptococci. Mutans are found in small numbers [22] and are more abundant in white spot lesions (WSLs) than in healthy regions. However, nonmutans streptococci still constitute most WSL bacteria. It has been shown that the initial members of microflora can singly dissolve enamel in the absence of mutans and lactobacilli [22]. In cavitated dentinal lesions, including rampant caries, mutans constitute about 30% of microflora, which indicates that mutans are linked with progressive carious lesions. Nevertheless, some studies have shown that the dentinal caries of mutans is less frequent in deep lesions, and lactobacilli, *Prevotella*, and bifidobacteria are more prominent. These results indicate that microflora on the tooth surfaces change as caries progresses [22]. *S. mutans* is Gram-positive cocci. The oral *S* spp. are normal flora but are also opportunistic and can cause dental caries [23]. They include *SM*, *S. sobrinus*, *S. rattus*, *S. cricetus*, *S. ferus*, *S. downei*, and *S. macacae*. Virulence has numerous factors that induce its demineralization [23] (Table 1).

## 6. Extracellular Polymeric Substance [24]

The extracellular polymeric substance [24] of the biofilm plays a pivotal role in maintaining the bacterial integrity and adhesion. New studies on the biology of EPS have reported several roles for the scaffold resulting from EPS, which are vital for the biofilm [25, 26]. Some of these roles include surface adhesion, spatial and chemical heterogeneity in biofilm, competitive or collaborative interactions, and improved resistance to antimicrobials [25]. The construction of EPS matrix relates to the existing substrates, production and secretion of e-materials, and shear forces. The key part of EPS content in oral biofilms which is related to caries is polysaccharides, especially the glucans derived from *SM* [27]. In addition to these, polysaccharides result from other bacteria (e.g., actinomycetes, *Streptococcus salivarius*, and *Streptococcus gordonii*), and combination compounds of starch glucan exist in this matrix. Further, this matrix contains eDNA [28] (proteins derived from bacteria with properties similar to amyloid), host GP which are able to participate in the scaffold with G such as glucan-eDNA compounds [29]. The function and structure of this class of extracellular polymers are still not known completely and require further studies. Glucans are composed of glucose components, which are connected by*α* 1–4 and *α* 1–6 glycosidic bonds and are created by the coordinated activity of streptococcal exoenzymes called glucosyltransferase [3, 27, 30]. Interestingly, these extracellular enzymes can bind to the tooth structure in the active state and produce glucans locally, thereby providing new binding sites for the bacteria. In addition to these, glucosyltransferase enzymes bind to other oral microorganisms (e.g., commensal *S*, actinomycetes, *L*, and even *Candida albicans*) and make them G producers [3, 27, 30].

The G produced on the tooth surface increase the surface bacterial aggregation. They also cause new interspecies interaction and increase cell-cell adhesion [3]. These extracellular polymers aggregate on different areas of tooth surface on the cell membrane, each having a complementary role in the EPS matrix formation and biofilm development. Some of these roles are surface adhesion, cell-cell adhesion, and cell cluster development found in the biofilm systems [31]. The biofilm matrix is developed three-dimensionally with biofilm maturation and production of extracellular polymers, covering cell clusters, building bridges between them, and creating a firmly divided structure. This heterogeneous structure resulting from EPS explains the presence of microbial clusters in various sizes and composition in the human oral biofilm [32–34]. The EPS sediment on the surface and its development affect the mechanical properties of biofilm like the binding strength of biofilm to the surface and its integrity [35]. A well-formed biofilm is hardly removed from the tooth surface and has reinforced viscoelastic properties that help it to remain on the surface during the shear stresses exerted by liquids [35]. The structure of the EPS matrix can be locally altered by dextranases, DNAs, and protolithic enzymes to create new binding sites for the following bacteria that have not been able to join the biofilm. The stiffness of the biofilm matrix is increased over time. The physicochemical properties of biofilm protect the buried bacteria by inhibiting the access of drugs and increasing their AM resistance. For instance, EPS can bind to cationic AM like CHX and AM peptides and prevent their permeation into the depth of biofilm, thus preventing their toxic effect [3, 34].

## 7. Fighting Dental Caries

**Figure 1:**
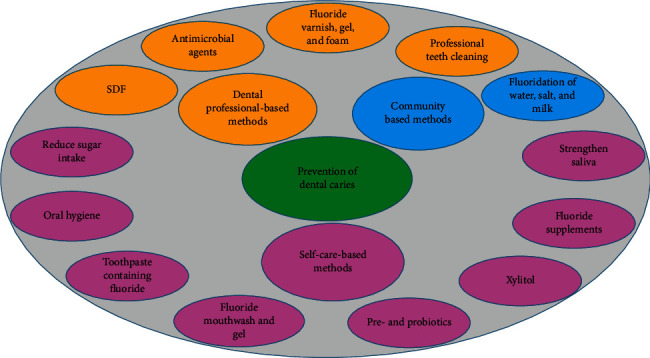
Prevention strategies of dental caries.

The caries-preventive strategies can be classified into three groups depending on the role and responsibility of the individuals doing them (Figure 1): (1) community-based strategies, (2) dental professionals-based strategies, and (3) individual-based strategies. The dental professionals-based strategies include professional tooth cleaning and use of varnish, fluoride gel and foam, fissure sealant, and antimicrobial agents. The individual-based (self-care) strategies consist of using fluoride toothpaste, fluoride supplements, fluoride mouthwashes, fluoride gels for personal use, chlorhexidine gels and mouthwashes for personal use, slow-release fluoride devices, oral health, diet control, and noncariogenic sweeteners like xylitol [36]. Recent studies (2015–2020) are mentioned in Table 2.

## 8. Communal Activity-Based Methods

### 8.1. Fluoridation of Water, Salt, and Milk

Fluoridation of drinking water has been used as an affordable practical way to reduce socioeconomic inequalities related to dental caries. Fluoridation of drinking water decreases dental caries by 30–50%, and termination of water fluoridation in conditions with inadequate alternative sources of fluoride increases caries by 18% [149]. Fluoridation of salt includes many advantages of water fluoridation, and consumers can choose to use it. The fluoride-containing salt will have different effects depending on the type of consumer. In high-consumption families, it can cause fluorosis [150]. Fluoridation of milk is beneficial for school children, especially for their permanent teeth. An advantage of this method is that it can be used in the high-risk group. Its consumption rate is also controllable. Moreover, individuals have the right to choose it or not. Fluoride in milk is absorbed more slowly than the fluoride in water due to the presence of calcium in milk [150, 151].

## 9. Dental Professionals-Based Methods

### 9.1. Professional Tooth Cleaning

Karlstad program can be used for biofilm control. In this program, the application of topical fluoride and diet, daily oral hygiene, and tooth cleaning with certain intervals are presented by a trained specialist. Further, this is done every two weeks in this program because studies have shown that the biofilm remaining for two to three weeks leads to the formation of WSL and caries. These intervals can be increased to three months in people with good oral health [152].

### 9.2. Use of Varnish, Gel, and Fluoride

The individuals aged <18 years are advised to use fluoride varnish every 3–6 months. Tooth caries due to the application of fluoride varnish has been reported to reduce 46% in permanent dentition and 33% in primary dentition [153, 154]. APF and NaF gels are used professionally. Studies have reported 26% reduction in caries in permanent dentition and 20% in primary dentition as a result of the application of 1% sodium fluoride twice per year. Fluoride foam has the same advantages and density as fluoride gel and releases the equivalent fluoride [153]. Fluoride exerts its anticaries effects by three different ways. In the first way, fluoride ion in dental tissues reinforces the fluorapatite deposition from the salivary phosphate and calcium ions. This insoluble deposition occupies the soluble salts including magnesium and carbonate lost during demineralization by the bacteria. This process makes the enamel more resistant to acid [9]. In the second way, caries becomes remineralized without the formation of the cavity using a similar process [9]. In the third way, fluoride ion has AM activity. At low concentrations, fluoride obstructs the construction of glycosyltransferase enzyme. Glycosyltransferase gets the glucose involved in the formation of extracellular polysaccharides and enhances the bacterial adhesion. The formation of extracellular polysaccharides is inhibited by limited bacterial metabolism during the meal time, which prevents the aggregation and maintenance of carbohydrates. Therefore, the duration of the attack of caries is limited to the period of eating and after it. High concentrations of fluoride ions (12000 ppm) are directly toxic for some oral microorganisms like *SM*. Fluoride has a wide range of activities and has long stability in the oral cavity. It reduces the acid production at 1–10 ppm concentration; it is bacteriostatic at 250 ppm and bactericidal at 1000 ppm [9]. The iodine group is bactericidal and has a wide range of antibacterial activities and short-term stability in the mouth [9]. Many studies have documented the efficacy of fissure sealant treatment in reducing occlusal caries in permanent dentition in children and adults with a high risk of caries [36]. The population-based studies have confirmed the cost-effectiveness of fissure sealant and its long-term effects. Since most caries in the current population occurs in the pits and fissures, fissure sealant seems to be beneficial [150].

### 9.3. Silver Diamine Fluoride (SDF)

Systematic reviews have recommended the application of SDF for termination or prevention of caries in children and adults and root caries in the aged people [155, 156]. This solution is used locally, and silver ions exert their antibacterial effects by breaking the bacterial membrane, denaturing the proteins, and preventing the DNA proliferation [157]. Silver and fluoride both have a key role in the termination of caries progress and sensitivity of tooth [158–161]. Silver reduces the demineralization speed and boosts the remineralization process [162].

### 9.4. Antimicrobial Agents

Numerous antimicrobial factors have been introduced to decrease the number of bacteria and disturb the biofilm structure. Dental decay is a biofilm associated disease which changes with regimen. Hence, changing the number of bacteria does not have a long-term effect on it. If a remarkable reduction does not occur in the consumption of fermentable carbohydrates, the microbiome in biofilm will adapt to the acidogenic environment and the uric acid produced by the cariogenic diets; thus, AM will have slight effect on the outcome of dental caries [9].

Principally, most antimicrobial agents used for prophylaxis contain a wide range of antimicrobials and provide a ground for the growth of opportunistic factors by eliminating the normal flora. Therefore, except for the consumers of fluoride toothpaste, all other chemical agents should not be used routinely in the daily schedule of the patients. These agents are used as an auxiliary aid when the routine prevention is not effective in individuals with a mental or physical disability, people with reduced salivary secretion, or cases with difficult mechanical removal of plaque such as conditions associated with orthodontic treatment, before and after oral surgeries, and frequent use of crowns [9]. Some antimicrobial agents along with their mechanism of effect are presented in Table 3.

## 10. Self-Care Methods for Caries Prevention

### 10.1. Reducing the Consumption of Fermentable Carbohydrates

The main reason for caries-induced dysbiosis is the overuse of fermentable carbohydrates [179]. The diet mechanism has significant effects on the impact of biofilm on dental caries. Frequent consumption of foods having sucrose changes the biofilm from a noncariogenic state to a cariogenic state. Established biofilm, which is repeatedly exposed to sucrose, quickly makes acids from it, thereby creating an acidic environment [22]. Dental caries is mostly caused by the frequency of sucrose consumption, not its amount [22]. The commensal bacteria use the sugar and make acid when a person has a low sugar diet; however, pH is quickly recovered by the mechanisms present in mouth. Frequent consumption of sugars disturbs the balance, and fully reciprocal acidogenic and aciduric strains appear in pathogenic amounts [180, 181]. WHO has seriously recommended restraining the intake of free sugars to <10% of the entire energy intake to prevent weight gain and dental caries [182]. This refers to the <50 g/day consumption of free sugars. Natural sugars such as sugars in honey and added monosaccharides to the foods are free sugars [182]. Cohort studies with quality evidence have reported 15% sugar as the moderate level. Other studies have also recommended the reduction of free sugars to 5% of total energy. Sugar consumption below 5% seems unlikely to cause any caries [183]. Diet with high amount of sugar causes the aggregation of acidogenic and acid-tolerant bacteria and protects them by increasing the production of EPS [27, 174]. Indirect evidence shows caries reduction by decreasing the intake of free sugars. For example, a significant decrease of caries during five years has been reported in the Iraqi children with reduced sugar intake due to the sanctions of the United Nations [184]. Starch as well as sucrose is known as a cariogenic agent. Starch's metabolism causes the long-term acidity in the pits and interdental spaces vulnerable to decay [180].

## 11. Oral Hygiene

The tooth surfaces free of biofilm are not decayed. Patients should regularly eliminate biofilm by brushing with fluoride-containing toothpaste and dental floss [9, 159]. The oral biofilm composition changes over time following oral hygiene by regular brushing twice a day, and oral microbiome is maintained in the healthy state [160]. The brushing does not remove oral bacteria totally but rather eliminates them from the tooth surface. A massive amount of them is removed from the mouth after swallowing and/or rinsing after brushing and flossing; however, an enough number of them stay for proliferation. Cleaning and exposing to oxygen may kill anaerobic organisms; however, no species is eliminated. Accurate mechanical tooth cleaning disrupts the dental biofilm and cleans tooth surface. While all bacteria that constitute established biofilm still do not exist, most of them are not able to bind to the clean tooth surface [9]. Brushing and flossing are advantageous in that they do not destroy the oral normal flora. Frequent mechanical removal of biofilm does not cause the risk of opportunist infection but rather changes the biofilm's composition. Patients with good oral hygiene have a high percentage of *S. mitis* or *S. sanguis* in their teeth biofilm and have a smaller amount of cariogenic bacteria than the more developed biofilm having a high amount of *S. mutans* [9]. Nevertheless, it should be emphasized that the former concepts of plaque removal or accurate control of another plaque are not considered for the management of caries. Proper oral hygiene is certainly important for biofilm control, but systemic review studies have shown that mechanical removal of biofilm alone, in the absence of fluoride, is not enough to manage caries [158].

### 11.1. Fluoride Toothpaste

One of the main causes of caries reduction in developed countries is the extensive application of fluoride TP. Easy and extensive application, low cost, and cultural acceptance have turned fluoride toothpaste into an ideal method for the promotion of general health, and brushing twice a day using fluoride toothpaste has been recommended as a powerful preventive strategy [185].

### 11.2. Fluoride Supplements

Fluoride supplements are capable of decreasing dental caries by about 20–30% and apply to populations with no access to fluoridated water. These supplements, due to the risk of fluorosis, should be used cautiously and can be used in kids who are at high risk of dental caries and committed parents [153].

### 11.3. Fluoride Mouthwashes and Gels for Personal Use

Fluoride mouthwashes with 0.2 and 0.05% concentrations are available for daily and weekly use and have been reported to prevent caries by 26%. The fluoride gels for personal use are available as APF, neutral NaF, and stannous fluoride and have been found to reduce caries by 32% in communities with inadequate fluoridated water [36, 153].

### 11.4. Noncariogenic Sweeteners

Numerous studies have reported the caries-preventive effect for xylitol and to a lesser extent for sorbitol. Xylitol can decrease biofilm [186]. Xylitol is a five-carbon sweetener that has beneficial effects on oral health. Most clinical studies have shown that daily consumption of ≥5 g xylitol gum is efficacious in decreasing caries [187, 188]. Use of xylitol decreases the *SM* amount [189]. Different mechanisms that can reduce mutans are growth inhibition, plaque reduction, oral pH increase, reduction of sticky polysaccharides produced by mutants, and inhibition of stress proteins [48, 58, 60, 92, 98, 108, 186–190].

### 11.5. Strategies to Boost Saliva

Salivary secretion plays a pivotal role in preventing dysbiosis and maintaining oral health. In addition to the mechanical deletion and buffering capability of saliva, it has enzymes, GP, salts, immunoglobulins, and AM peptides which help with the biofilm stability and control [162]. Although there are clear salivary stimulants, salivary induction is difficult in practice. In patients who are still able to produce saliva, local methods like increasing water consumption and chewing gums and salivary alternatives regularly can enhance saliva making. The moisturizers and enzymes in the products can decrease the symptoms of patients with dry mouth and maintain healthy biofilm [4, 191, 192].

### 11.6. Prebiotics and Probiotics

Prebiotics are oligosaccharides and nutritional fibers which contribute to useful bacteria's growth while probiotics are defined as living microbes which have advantages for patients [155]. There are not many studies on prebiotics in dentistry, while there are more studies on Arg. Arg is an aminoacid that is found in saliva. The bacteria in healthy biofilm could use Arg through ADS. There is high activity of ADS in noncarious places rather than places with caries lesions [157, 193]. Moreover, studies [157, 194] have shown that adding Arg to fluoride TP can enhance the ADS activity, increase the pH, and help to make a healthy biofilm [157, 175, 193].

The analysis of clinical trials has shown the synergistic effect of arginine and fluoride on the primary caries [176]. Another interesting aspect of prebiotics is breastfeeding. Breast milk is rich in complex biotic oligosaccharides. Systemic studies have shown that breastfeeding in the first year has protective effects against ECC, and bottle-fed newborns had four times more caries than breastfed newborns [195, 196]. Recently, application of probiotics to fight harmful bacteria has increased [156]. Probiotics are dietary supplements that are principally consumed through fermented vegetables, dairy products, and bread. Additionally, there are different forms of these materials such as tablets, drops, and lozenges that have been commercialized. Their mechanism is not known completely. They have effects on biofilms directly and on the immune system [197]. Studies have concluded that therapeutic probiotics can especially reduce the number of *S. mutans* [198]. However, there are very few studies on the effect of oral microbiome composition. Moreover, an increase in nonmutans streptococci and a decrease in the count of *S. mutans*, *Fusobacterium*, and *Prevotella* have been reported after using a supplement for 12 weeks [199]. In contrast, another study has found that the use of lozenges containing *Lactobacillus rhamnosus* and *Brevibacillus brevis* has no significant effect on the oral microbiome in adults after 4 weeks. These conflicting results may be partly due to different molecular and sequencing technologies used in various studies. The results of these studies have shown a 33% reduction in caries in preschool children [200]. Nevertheless, there is insufficient evidence for their overall recommendation. Probiotic treatment for oral health is one of the highly advanced emerging concepts.

## 12. Conclusion

One of the basic aspects of general health is oral health. Oral diseases cause many problems for individuals and society. They are very common all over the world. While there are effective methods for and adequate information about the prevention of oral diseases, they are still one of the most prevalent health problems. The best strategy to fight caries is the use of a combination of community-based strategies, oral health professionals-based strategies, and self-care strategies. The most important strategies to fight against caries are paying attention to the oral health in macroeconomic policies of countries to create a healthy society, changing the attitude of dental professionals from a treatment-centered approach to a preventive approach, and empowering the individuals by enhancing their knowledge, attitude, and performance in line with maintaining and promoting their oral health.

## 13. Future Directions

Despite many attempts made to control caries, these efforts have not been effective in controlling caries. Further studies are suggested to identify the confounding factors of biofilm such as natural antimicrobial materials that, unlike antibiotics, do not cause bacterial resistance and are cheap and available to everyone. Moreover, more studies are required on probiotics and production of *S. mutans* strains that are not able to produce lactic acid by recombinant DNA technology or other bacteria that competitively eliminate the microbial cariogenic agents of the oral cavity and manufacture of slow-release devices for antimicrobials such as fluoride in the oral cavity and silver nanoparticle pins that prevent caries by releasing antimicrobial agents. Furthermore, we suggest conducting future studies on the production of caries vaccine to induce biologic defense against caries in the oral cavity and creation of protective layers on the tooth that act beyond the current sealants in terms of gear, duration, and caries prevention.

## Figures and Tables

**Table 1:** Virulence factors of *SM*.

Property	Description
Making acid	Capability to make lactic acid

Aciduricity	Capability to tolerate low pH

IPS	Capability to consume IPS to continue making lactic acids with the lack of fermentable carbohydrates

EPS	Making matrix of the biofilm

**Table 2:** Recent strategies in dental caries prevention.

Treatment	Type of study	Methods	Outcomes	Ref/year
Stannous fluoride products	In vivo	The groups were as follows: the test group using stannous fluoride products and the control group.	The analysis showed no effect of stannous fluoride products.	[37]/2018

Sodium fluoride dental protective agent combined with pit and fissure sealant	In vivo	The groups were as follows: in the control group pit and fissure sealant was used, and in the test group sodium fluoride tooth protector joined with pit and fissure sealant was used.	The combined group had better results.	[38]/2019

GI and RBS	In vivo	The groups were as follows: GI and RBS.	Groups were the same in the survival of GI and RBS.	[39]/2017

Ozone, sealant, and fluoride varnish	In vivo	The groups were as follows: (1) control; (2) fluoride varnish; (3) sealant; (4) ozone.	The use of fissure sealant, fluoride varnish, and ozone is suggested for prevention of occlusal pit and fissure caries.	[40]/2016

Silver diamine fluoride (SDF)	In vivo	SDF (38%) or Pl was applied topically. The primary outcome was caries arrest (Nyvad criteria).	Topical 38% SDF was effective in arresting cavities.	[41]/2018

RBS and GI	In vivo	The groups were as follows: RBS, GI sealant, and control.	The RBS was higher than the GI sealant in prevention of caries.	[42]/2018

F coating joined with PRF or PTS	In vivo	The groups were as follows: group A, PTS; B, PRF; C, 0.5% F coating + pit and fissure sealing; D, 0.5% fluoride + preventive resin filling; E, control.	Groups C and A had a certain effect on prevention of dental caries, but group D was better.	[43]/2018

*Lactobacillus paracasei*	In vivo	The groups were as follows: probiotic milk or control (standard milk).	Probiotic milk reduced salivary *S. mutans* count.	[44]/2018

MIV and MIPP	In vivo	The groups were as follows: FTP, using MIV + MIPP application, and control.	Groups were the same in ICDAS scores and EDI sum.	[45]/2018

CXT and FJ	In vivo	The groups were as follows: CXT or FJ.	GIC sealants were effective in preventing caries.	[46]/2018

PTS and FV	In vivo	The groups were as follows: resin-based fissure sealant or FV was applied.	Groups were the same in caries prevention.	[47]/2017

Xylitol-containing chewing gum	In vivo	The groups were as follows: in the test group xylitol gum + oral health education were used, and in control group oral health education alone was used.	Both groups had a reduction in the caries rate.	[48]/2017

MIn	In vivo	The groups were as follows: CE or MIn.	MIn group was better in prevention of caries.	[49]/2019

PTS combined with fluorine protective paint	In vivo	The groups were as follows: control, PTS, and PTS + fluorine protective paint.	Pit and fissure sealant + fluoride protective paint can decrease the incidence of dental caries.	[50]/2019

MIn	In vivo	The groups were as follows: HE, MIn, and MI + RA.	MIn group had higher preventive effects against caries than HE group.	[51]/2017

TiF_4_ varnish	In vivo/in situ	The groups were as follows: TiF_4_ varnish, Duraphat, Pl varnish, and no treatment.	F-varnishes showed caries-preventive effect.	[52]/2019
Silver NP + PTS	In vivo/in situ	The groups were as follows: conventional and silver NP combined with PTS.	Silver NP mixed sealant was more effective than conventional sealant in reducing tooth demineralization.	[53]/2017

Fluoride varnish	In vivo	The groups were as follows: fluoride varnish or Pl.	Quarterly applications of fluoride varnish were not effective in preventing development of dental caries.	[54]/2016

Different fluoride regimens	In vivo	The groups were as follows: G1: control group, fluoride (F) TP (1450 ppm); G2: FTP (1450 ppm) + 0.2% F oral rinse; G3: TP (5000 ppm).	The recommendation was application of FTP (5000 ppm F) or oral rinse (0.2% NaF) + usual TPs.	[55]/2019

Moisture tolerant RBS and GIS	In vivo	The groups were as follows: moisture tolerant RBS or GIS was placed on one side of the mouth, and the other one was placed on the opposing side. DIAGNOdent readings were taken.	Both materials were effective in arresting enamel caries.	[56]/2019

Fluoride varnish Duraphat	In vivo	The groups were as follows: fluoride varnish and control.	Fluoride varnish Duraphat was effective in decreasing caries incidence.	[57]/2019

Low-dose xylitol chewing gum	In vivo	The groups were as follows: xylitol and polyols.	Xylitol group showed a significantly lower increment of dental caries.	[58]/2017

RBS with and without F	In vivo	The groups were as follows: sealants with or without fluoride and control.	The effects of the sealants were similar.	[59]/2018

Xylitol and polyol chewing gum	In vivo	The groups were as follows: xylitol chewing gum, polyol chewing gum, and control group.	Xylitol-containing chewing gum was effective in decreasing caries incidence.	[60]/2018

Oral health education (OHE) and FV	In vivo	The groups were as follows: control, OHE, and OHE + FV.	OHE or OHE + FV reduced the caries incidence.	[61]/2016

FM and D	In vivo	The groups were as follows: participants brushed their teeth with either a D (1150 ppm) or a Pl D without F and either daily application of FM (220 ppm) or not.	FM was effective in remineralization.	[62]/2018

Biannual treatment with FV	In vivo	The groups were as follows: standard yearly intervention with or without FV.	Biannual treatment with FV was not effective in preventing dental caries.	[63]/2017

STB and S	In vivo	The groups were as follows: STB, CR sealant, and ART-GIC sealant.	The groups were the same in preventing caries.	[64]/2015

SDF 12% and SDF 38%	In vivo	The groups were as follows: 12% SDF applied yearly, 12% SDF applied twice a year, 38% SDF applied yearly, and 38% SDF applied twice a year.	Higher concentration or frequency of SDF had more effect in arresting active tooth caries.	[65]/2018

FV and peptide P_11_-4	In vivo	The groups were as follows: P_11_-4 + FV or FV.	P_11_-4+FV was effective in early carious lesions.	[66]/2018

CHX/thymol V or FV	In vivo	The groups were as follows: three-time monthly use of CHX/thymol varnish or semiannual use of FV + semiannual use of Pl V.	The groups were the same in dental caries development.	[67]/2015

FM, EO, and CHX oral rinses	In vivo	The groups were as follows: FM; EO; CHX; control (saline).	FM and CHX had more effect than EO mouth rinse.	[68]/2015

F, CPP-ACP, IR	In vivo	The groups were as follows: A, control (blank); B, control (Irr); C, Irr + F; D, Irr + CPP-ACP, E, Irr + CPP-ACP + F; F, Irr + IR; G, Irr + IR + F; H, Irr + IR + CPP-ACP.	IR + CPP-ACP, IR + F, CPP-ACP + F, and IR were the best effective methods to prevent Irr-dentin-destructions.	[69]/2019

TP containing Arg	In vivo/in vitro	Individuals wearing a dental device: the studies stages were lead-in, Arg-free, washout, and Arg-active stages.	Arg-containing TP can significantly decrease the LA construction.	[70]/2017

Varnish containing chlorhexidine	In vivo	The groups were as follows: Cervitec Plus® or Pl varnishes.	Application of Cervitec Plus® had a significant advancement in patients' oral health.	[71]/2018

Herbal extracts (Tulsi and Black myrobalan) and sodium fluoride	In vivo	The groups were as follows: (1) FM, (2) Tulsi mouth rinse, and (3) Black myrobalan mouth rinse.	Herbal mouth rinses could be tried as an anticaries agent for dental caries.	[72]/2018

FV	In vivo	FV applied every three months.	The use of fluoride varnish every three months prevented the incidence of caries.	[73]/2019

Infiltrant application	In vivo	The groups were as follows: icon infiltrant (DMG) and PFS (Alpha Seal-DFL).	The infiltrant was effective in preventing the caries progression comparable with the conventional sealant.	[74]/2017

Fluoridated milk	In vivo	The groups were as follows: fluoridated milk and nonintervention.	Consumption of fluoridated milk could significantly (34%) reduce the caries.	[75]/2018

PRG filler-containing sealant placed with a self-etching primer/adhesive	In vivo	The groups were as follows: self-etch primed sealant (BeautiSealant, Shofu) or the etch and rinse sealant (Seal it, Spident).	The groups were the same in caries prevention.	[76]/2018

High-fluoride toothpaste	In vivo	The groups were as follows: 5,000 ppm F toothpaste or 1,450 ppm F toothpaste.	High-fluoride toothpaste had more effects than control toothpaste in preventing caries.	[77]/2019

FV	In vivo	The groups were as follows: FV or Pl.	FV application was not effective in children.	[78]/2018

MIn	In vivo	The groups were as follows: HE and MIn.	MIn had more effect than HE in reducing caries.	[79]/2018

Hydrophilic F-releasing sealant and ACP sealant	In vivo	The groups were as follows: Aegis™ or Embrace WetBond™ sealant.	Aegis™ was more effective than Embrace WetBond™ sealant as Aegis™ demonstrated lower caries scores.	[80]/2019

School-based fluoride varnish program	In vivo	Volunteers used FTP at home.	The school-based fluoride varnish program prevented progression of caries.	[81]/2016

Topical F	In vivo	The groups were as follows: (1) annual use of SDF solution (30%); (2) three-time use of SDF (30%) per week; (3) three-time use of 5% FV per week.	Yearly use of SDF solution had more effect than three-time use of FV or SDF solution.	[82]/2018

Nutrition and hygiene education	In vivo	The groups were as follows: intervention and control.	The education intervention reduced the progression of caries.	[83]/2018

Organoselenium-containing pit/fissure sealant (DenteShield™ (DS)) and UltraSeal™ XT Plus (UXT)	In vivo	The groups were as follows: DS and UXT.	The groups had the same results for Caries prevention.	[84]/2019

Fluoride TP	In vivo/in vitro	Volunteers used FD or not.	FD group had lower demineralization.	[85]/2016

Toothpastes with fluoride and hydroxyapatite	In vivo	The groups were as follows: toothpastes with hydroxyapatite and fluoride.	Observation group had significantly higher (*p* > 0.05) acid resistance compared with the group of patients using fluoride toothpaste.	[86]/2018

Ordinary and PB cake (*Bacillus coagulans*)	In vivo	The groups were as follows: (1) 1-week consumption of PB cake, then 4-week washout period, and 1-week consumption of regular cake; (2) consumption of the cakes was reversed.	The addition of PB bacteria led to a slight increase in the number of *SM* bacteria in the saliva.	[87]/2019

Ordinary TB and an interactive power TB	In vivo	The groups were as follows: power TB with Bluetooth technology or an ordinary handy TB.	An interactive power TB was more effective in plaque removal versus a handy TB.	[88]/2019

Food enriched with probiotics	In vivo	The groups were as follows: PB milk and standard milk.	The groups were the same in the incidence of caries.	[89]/2018

Resin infiltration	In vivo	The groups were as follows: 1) FTP + flossing + infiltration; 2) control group (FTP + flossing).	Infiltration group had better results than control group.	[90]/2018

GI sand resin s	In vivo	The groups were as follows: GIS and RS.	GISs presented effective prevention of caries development.	[91]/2016

PB yogurt and gums with xylitol	In vivo	The groups were as follows: PB yogurt or gums with xylitol.	The groups were the same in reduction of *SM* counts.	[92]/2017

Fissurit FX sealant and Grandio Seal nanofilled fissure sealant	In vivo	The groups were as follows: Fissurit FX sealant and Grandio Seal nanofilled fissure sealant.	Fissurit FX and Grandio Seal pit and fissure sealants were similar in caries prevention.	[93]/2019

Photodynamic therapy and US	In vivo	The groups were as follows: PDT with MB and US.	PDT or US postponed side effects.	[94]/2018

Fluoride varnish or fluoride mouth rinse	In vivo	The groups were as follows: semiannual fluoride varnish applications (FV) and fluoride mouth rinses once per week (FMR).	The groups had the same results in dental caries progress.	[95]/2016

PB and normal milk	In vivo	The groups were as follows: PB milk and standard milk.	Long-term drinking of probiotic milk may decrease caries progress.	[96]/2016

Intensive FV	In vivo	The groups were as follows: 3 applications of FV in 2 weeks and extra applications at 1 and 3 months; FV treatment twice a year.	The intensive FV application had no adequate effect to prevent dental caries.	[97]/2018
Erythritol	In vivo	The groups were as follows: erythritol, xylitol, or sorbitol (control) group.	Erythritol consumption had caries-preventive effect.	[98]/2016

Interdental cleaning device	In vivo	The groups were as follows: manual toothbrush + mechanical interdental device or manual toothbrush alone.	The combination group had a superior plaque removal compared to manual brushing alone.	[99]/2018

FTP containing zinc ions	In situ	The groups were as follows: F, F/ZN/phytate, F/Zn, and F Pl.	Phytate had slight effect on capability of fluoride to prevent more advanced lesion demineralization. Moreover, zinc ions had no bad effect on fluoride ability.	[99]/2018

High-fluoride varnish	In vivo	The groups were as follows: differing frequencies of Duraphat varnish application.	Periodic application of fluoride varnish could be useful in prevention of white spots.	[100]/2016

Fluoride varnish	In vivo	The groups were as follows: control and use of FV (every 3 or 6 months).	Results suggested using FV with three-month intervals for prevention of caries.	[101]/2019

Fluoride varnish	In vivo	The groups were as follows: (1) dental hygiene + FTP and one-time use of three varnishes: Fluor Protector S, Elmex® fluid, or control (Pl).	FV application had no extra protective benefit.	[102]/2016

Toothpaste with nanosized sodium hexametaphosphate	In vivo/in vitro	The groups were as follows: conventional fluoride TP, fluoride TP (1100 ppm), fluoride TP (1100F + micro HMP), and fluoride TP (1100F + nano HMP).	1100F/HMPnano revealed a superior protective effect against enamel demineralization.	[15]/2019

Three different compositions of topical fluoride varnishes	In vivo	The groups were as follows: FV having CPP-ACP; FV having xylitol; FV with 0.9% difluorosilane.	FV having CPP-ACP showed higher decrease in *SM* count.	[103]/2019

CHX MR, combination MR, and green tea extract MR	In vivo	Volunteers used different MR.	Green tea mouth rinse was effective in prevention of caries.	[104]/2017

Povidone-iodine (PI), CHX, or FV (fluor protector)	In vivo	The groups were as follows: PI, CHX V, or FV and control.	Fluoride varnish showed higher decrease in S. *mutans* count.	[105]/2017

Probiotic milk and fluoride mouth rinse	In vivo	The groups were as follows: probiotic milk and fluoride mouthwash.	Groups were the same in reduction of *S. mutans* and PI scores.	[106]/2019

Topical fluorides	In vivo	The groups were as follows: group 1, 30% SDF solution yearly; group 2, 30% SDF solution per week; group 3, 5% FV per week.	Application of SDF had more effect on arresting caries than FV.	[107]/2016

Milk sweetened with xylitol	In vivo	The groups were as follows: (a) xylitol milk, 8 g/200 ml, one time daily; (b) xylitol milk, 4 g/100 ml, two times daily; (c) sorbitol milk, 8 g/200 ml, one time daily; (d) sorbitol milk, 4 g/100 ml, two times daily; or (e) sucrose milk 8 g/200 ml, one time daily.	There were no significant differences in caries incidence between groups.	[108]/2016

Probiotic chewing tablets	In vivo	The groups were as follows: the test group got chewing probiotic tablet and the Pl group got the same tablets without bacteria.	Probiotic chewing tablets could be helpful in reducing caries.	[109]/2015

Fluoride TP	In vivo	The selected product was brushed twice daily for 4 months.	Clinpro 5000, Clinpro Crème, and MI paste Plus all could be helpful in reducing white spot lesions.	[110]/2019

Probiotic lozenge	In vivo	The groups were as follows: probiotic lozenge and Pl lozenge.	Probiotic group had significantly lower *S. mutans*.	[111]/2019

Self-etching adhesives having an AB agent and/or F	In vitro/in vivo	The groups were as follows: fluoride-containing (One-Up Bond F Plus, OP), MDPB and fluoride-containing adhesive (Clearfil Protect Bond, PB).	The AB group had lower demineralization adjacent to restorations.	[112]/2015

CPP-ACP and xylitol gum	In vivo	The groups were as follows: gum containing CPP-ACP and xylitol.	Both gums increase saliva's properties.	[113]/2017

Resin infiltration	In vivo	The groups were as follows: test group lesions were treated with resin infiltration + 5% topical NaF application and control group with 5% NaF alone.	Resin infiltration was more effective in reducing the development of initial proximal enamel lesions compared with the other group.	[114]/2018

Salt fluoridation	In vivo	The groups were as follows: salt containing fluoride and control.	Salt containing fluoride was more effective in prevention of caries.	[115]/2018

Sodium fluoride varnish	In vivo	The groups were as follows: intervention group (fluoride varnish) and control group.	Significant caries reversal was seen in primary dentition after intensive fluoride application after 1 year of study.	[116]/2017

Sour cherry extract	In vivo	The groups were as follows: gum with cherry extract or control.	Sour cherry extract may have effect on prevention of caries.	[117]/2018

Arginine-containing TP	In vivo/in vitro	The groups were as follows: fluoridated TPs (FD) and arginine-containing fluoridated TPs (AFD).	AFD had an anticaries effect like that of ordered fluoridated TPs.	[118]/2018

PBM of major salivary glands	In vivo	The groups were as follows: continuous mode LED light, pulsed mode LED light, and control group.	Results suggested that PBM of salivary glands reduces risk of caries.	[119]/2020

Herbal mouthwash	In vivo	The groups were as follows: herbal mouthwash, chlorhexidine mouthwash, or Pl mouthwash.	The effectiveness of herbal mouthwash in decreasing plaque formation was similar to chlorhexidine.	[120]/2018

Filling intervention health education	In vivo	The groups were as follows: intervention group receiving filling of teeth; and health education group.	Intervention group had better results.	[121]/2015

CHX and F MR	In vivo	The groups were as follows: (a) CHX (0.12%) + NaF (0.2%); (b) NaF (0.2%); (c) CHX (0.12%); (d) control.	Groups a and c had similar plaque formation.	[89]/2018

Resin infiltration	In vivo	The groups were as follows: resin infiltration or control.	Progression of caries was significantly higher in control versus infiltration group.	[122]/2018

Probiotic *Lactobacillus reuteri*	In vivo	The groups were as follows: probiotic lozenges and Pl lozenges.	Probiotic lozenges reduced bacterial counts significantly.	[123]/2018

GIC sealant	In vivo	The groups were as follows: sealant application with or without extra light curing.	Caries prevention in both groups was similar.	[124]/2019

Propolis dental varnish	In vivo	Propolis varnishes were used in different concentrations (1%, 2.5%, 5%, and 10%).	Propolis V has AM activity.	[125]/2020

Resin infiltration	In vivo	The groups were as follows: infiltration and control.	Resin infiltration was more effective in reducing caries progression.	[126]/2016

GIS covered with resin-based agents	In vivo	Fuji VII was used and covered with G-Coat Plus or Heliobond.	The results were the same in both groups in incidence of caries.	[127]/2017

CPP-ACP	In vivo	The groups were as follows: stannous F gel (0.4%) with or without CPP-ACP.	CPP-ACP was not effective in decreasing caries development.	[128]/2015

New sealant	In vivo	The groups were as follows: Select Defense™ sealant; control.	Test group had lower incidence of WSLs.	[129]/2016

Atraumatic restorative treatment by chlorhexidine: disinfection or incorporation	In vivo	The groups were as follows: group (a) CHX having GIC; group (b) CHX; group (c) regular GIC.	Both chlorhexidine disinfection and incorporation showed higher efficacy in inhibiting residual microbes compared to conventional ART.	[130]/2017

Fluoride-releasing resin composite	In vitro	The groups were as follows: intervention group (F having adhesive resin) and control.	The materials used in test group were not effective in prevention of WSL.	[131]/2017

Fluoridated milk	In vivo/in vitro	Volunteers used an intraoral appliance. They dipped it in fluoridated milk for 5 minutes and once every other day drank the same milk.	Drinking fluoridated milk once per day prevented enamel demineralization.	[132]/2018

Toothbrush with paste and Munident	In vivo	The groups were as follows: normal TP and Munident.	Munident (herbal) TP group had significantly lower *S. mutans*.	[133]/2017

Fluoride TP and GC Tooth Mousse	In vivo	The groups were as follows: fluoride TP, CPP-ACP crème, and fluoride TP + CPP-ACP crème.	All groups had the same results; combination groups did not have additive benefits.	[134]/2020

MIPP and Er: YAG laser	In vitro	The groups were as follows: (a) MIPP; (b) Er: YAG laser; (c) MIPP + Er: YAG laser; (d) saliva; (e) control.	Group c was the most effective group in the treatment of WSLs.	[135]/2020

Probiotic bacterium *Lactobacillus reuteri*	In vivo	The groups were as follows: probiotic lozenge and Pl lozenge.	Probiotic lozenges did not prevent progressing of WSL.	[136]/2016

RMGI cement varnish	In vivo	The varnish was applied to teeth.	Application of RMGI cement varnish could be useful in preventing WSLs.	[137]/2015

Probiotic *Streptococcus dentisani*	In vivo	The probiotic was applied in a buccoadhesive gel.	*S. dentisani* was able to buffer oral pH, especially after multiple dosing.	[138]/2020

Semiannual fluoride varnish application	In vivo	The groups were as follows: typical oral health program with or without FV twice a year.	Applications of FV + typical oral health program did not decrease caries progress.	[139]/2016

CPP-ACP	In vivo	The groups were as follows: test group receiving CPP-ACP paste monthly and control group.	Test group had lower WSL compared to the control patients.	[140]/2016

Peptide P_11_-4	In vivo	The groups were as follows: P_11_-4 or FV.	Application of P_11_-4 significantly reduced the size of early carious lesions. This reduction was higher than fluoride varnish application.	[24]/2020

CHX MR and neem MR	In vivo	The groups were as follows: group a: CHX MR; group b: neem MR; group c: control.	Both MR significantly decreased PI index.	[141]/2017

TiF_4_ V	In vivo/in vitro	TiF_4_, NaF (2.45% F), or control (Pl V).	TiF_4_ V was the only treatment able to improve enamel remineralization.	[142]/2017

Fluoride and sodium hexametaphosphate in toothpaste	In vivo/in vitro	TP having 1100 ppm F and 1100F + HMP1% and Pl.	TP containing HMP1% was more effective than TP containing 1100F in decreasing demineralization.	[143]/2015

Toothpaste Apadent Total Care medical nanohydroxyapatite	In vivo	Volunteers used Apadent Total Care toothpaste with nano-calcium hydroxyapatite.	Application of toothpaste with nanohydroxyapatite showed the improvement of all indices.	[144]/2016

Protective chlorhexidine varnish layer over resin-infiltrated proximal carious lesions	In vivo	The groups were as follows: in the test group infiltration + double layer of chlorhexidine varnish was used and in the control group only infiltration was used.	Results suggest application of chlorhexidine varnish layer on resin infiltration when surface had microcavitation.	[145]/2016

AgNO3 solution and FV	In vivo	The groups were as follows: (1) AgNO_3_ solution (25%) + FV; (2) SDF (38%) + Pl V.	Results suggest application of AgNO3/NaF for management of ECC.	[146]/2015

Tooth Mousse		The groups were as follows: CPP-ACP (daily) and control.	CPP-ACP reduced *Streptococcus mutans* in test group.	[147]/2016

Fluoride rinse		The groups were as follows: sodium F + amine F; control.	Application of fluoride rinse helps prevent demineralization.	[148]/2015

**Table 3:** Some antimicrobial agents and their mechanisms.

Antimicrobial agents	Combinations	Mechanisms	Ref
Antibiotics	Aminoglycosides	Inhibiting protein synthesis	[9, 163]
	Glycopeptides	Interfering with the construction of the cell wall	
	Penicillins	Interfering with the construction of the cell wall	
	Quinolones	Preventing DNA replication and transcription	
	Rifamycins	Inhibiting transcription	
	Tetracyclines	Inhibiting protein synthesis	
	Actinobolin	Obstructing protein synthesis	

AMEs^*∗*^	Lysozyme	Catalyzing glycosidic bond hydrolysis in bacterial cell wall peptidoglycans	[9, 164, 165]
	Acylase	Quorum-quenching	

AMPs^*∗*^	Natural	Pore construction in membrane	[19, 166]
		Inhibition of metabolism	
	Synthetic AMPs	Pore construction in membrane	
		Inhibition of metabolism	

Cationic compounds	Chitosan	Disruption of cell membrane	[9, 167]
	Chlorhexidine	Disruption of cell walls	
	Poly(*ε*-lysine)	Destroying the outer membrane	
	QACs^*∗*^	Interference with enzymes	

Metal and metal oxides	Ag nanoparticles	Making oxidative stresses	[168–170]
		Disabling enzymes of bacteria	
		Affecting the permeability of the cell membranes	
	Cu nanoparticles	ROS^*∗*^ construction	
		Lipid peroxidation in membranes	
	TiO_2_ nanoparticles	ROS^*∗*^ construction Disrupting phosphorylation	
	ZnO nanoparticles	Making ROS^*∗*^	
		Making membrane more permeable	

Other noncationic compounds	Nitric oxide givers	Making nitrosative stresses Making oxidative stresses	[9, 13, 171]
		Disruption in signaling	
	Triclosan	Disrupting synthesis of fatty acid	

Natural materials	Tea	Disruption of membrane	[26, 172, 173]
		Inhibiting salivary amylase activity	
	Propolis	Interaction with bacterial membrane	
	Cranberry	Inhibition of biofilm formation	

Amino acids	Arginine	Keeping a well dental biofilm	[70, 118, 157, 174–177]

Antioxidants	Antioxidants	Disrupting proteins, decreasing bacterial EPS	[178]

^*∗*^AMEs: antimicrobial enzymes; AMPs: antimicrobial peptides; QACs: quaternary ammonium compounds; ROS: reactive oxygen species.

## Abbreviations

AB: Antibacterial
ACP: Amorphous calcium phosphate
ADS: Arginine deiminase system
AM: Antimicrobials
AMEs: Antimicrobial enzymes
AMPs: Antimicrobial peptides
Arg: Arginine
ART-GIC: Atraumatic restorative treatment-high-viscosity glass-ionomer cement
B: Bifidobacterium
CE: Conventional oral health education
CHX: Chlorhexidine
CPP-ACP: Casein phosphate polypeptide-amorphous calcium phosphate
CR: Composite resin
CXT: Clinpro XT
Dl: Deciliters
E: Extracellular
ECC: Early childhood caries
EDI: Enamel decalcification index
EO: Essential oil
EPS: Extracellular polysaccharide production
*F*: Fluoride
*J*: Fuji IX GP FAST
FN: *Fusobacterium nucleatum*
FV: Fluoride varnish
G: Glucans
GI: Glass ionomer
GIS: Glass-ionomer sealant
GP: Glycoproteins
HE: Health education
ICDAS: International caries detection and assessment system
IPS: Intracellular polysaccharide production
IR: Infiltration resin
Irr: Irradiation
*L*: *Lactobacterium*
LA: Lactic acid
MB: Methylene blue
MIn: Motivational interviewing
MIPP: MI paste plus
MR: Mouth rinse
NP: Nanoparticles
*P*: *Prevotella*
PB: Clearfil Protect Bond
PBM: Photobiomodulation
PFS: Pit and fissure sealing
*PG*: *Porphyromonas gingivalis*
*P.Int*: *Prevotella intermedia*
Pl: Placebo
PRF: Preventive resin filling
PRG: Prereacted glass
QACs: Quaternary ammonium compounds
RA: Risk assessment
RBS: Resin-based sealants
Ref: Reference
ROS: Reactive oxygen species
RMGI: Resin-modified glass ionomer
*S*: *Streptococcus*
SDF: Silver diamine fluoride
*SM*: *Streptococcus mutans*
STB: Supervised tooth brushing
TB: Toothbrush
TP: Toothpaste
US: Ultrasonic scaler
*V*: Varnish
WHO: World Health Organization
WSLs: White spot lesions
Xy: Xylitol.
